# Circle of Willis Morphology in Primary Intracerebral Hemorrhage

**DOI:** 10.1007/s12975-022-00997-7

**Published:** 2022-02-19

**Authors:** Mangmang Xu, Qian Wu, Yajun Cheng, Shuting Zhang, Wendan Tao, Shihong Zhang, Deren Wang, Ming Liu, Bo Wu

**Affiliations:** Center of Cerebrovascular Diseases, Department of Neurology, West China Hospital, Sichuan University, Chengdu, Sichuan Province China

**Keywords:** Circle of Willis, Primary intracerebral hemorrhage, Cerebral small vessel disease, ICH etiology, Cerebral microbleeds

## Abstract

We aimed to study the distribution of Circle of Willis (CoW) morphology and its association with intracerebral hemorrhage (ICH) etiology and cerebral small vessel disease (CSVD) burden. Patients with primary ICH who had brain MRIs were consecutively enrolled between March 2012 and January 2021. CoW morphology, CSVD features and the combined CSVD burden (including global CSVD burden, total hypertensive arteriopathy [HA] burden, and total cerebral amyloid angiopathy [CAA] burden) were assessed. CoW morphology included poor CoW (defined as CoW score 0–2), incomplete CoW, and complete fetal-variant of the posterior communicating artery (CFPcoA). Among 296 patients enrolled, 215 were included in the analysis. There was no significant difference among HA-, CAA-, and mixed-ICH in each CoW morphology. Exploratory subgroup analyses suggested that poor CoW was associated with a greater incidence of HA-ICH and low incidence of mixed ICH in patients aged < 60 years, while mixed ICH occurred more frequently in patients with CFPcoA, especially in those without hypertension history (all *p* < 0.050). Additionally, incomplete CoW was correlated with a larger incidence of lacunes (adjusted *OR* [adOR] 2.114, 95% *CI* 1.062–4.207), microbleeds ≥ 5 (adOR 2.437, 95% *CI* 1.187–5.002), and therefore the combined CSVD burden (adOR 1.194, 95% *CI* 1.004–1.419 for global CSVD burden, adOR 1.343, 95% *CI* 1.056–1.707 for total CAA burden), independent of modifiable vascular risk factors, but not age and sex. The CoW might therefore have a potential impact on ICH etiology and is associated with a greater CSVD burden. Our findings are novel, and need to be verified in future studies.

## Introduction

Hypertensive arteriopathy (HA) and cerebral amyloid angiopathy (CAA) are two common forms of cerebral small vessel diseases (CSVD), which are responsible for the major causes of primary intracerebral hemorrhage (ICH) [1, 2]. Despite the devastating consequences of primary ICH, the mechanisms underlying ICH are poorly understood. It is well established that hypertension is the most crucial risk factor for HA-ICH [3, 4], and an increasing burden of CSVD features are associated with ICH occurrence [5] and recurrence [6, 7]. The distribution of CSVD features, such as lacunes, white matter hyperintensities (WMH) of presumed vascular origin, cerebral microbleeds (CMBs), cortical superficial siderosis (cSS), and enlarged perivascular spaces (EPVS) [3], is distinctive in severe HA and CAA [8], making them useful for identifying the pathological mechanisms of ICH.

The Circle of Willis (CoW) varies among the general population, and it has been hypothesized that cerebral autoregulation could be affected by the absence of communicating arteries of CoW [9]. A previous study demonstrated that an incomplete posterior CoW might be a factor in triggering hypertension [10]. Additionally, CoW morphology plays a fundamental role in CSVD features. In healthy individuals, incompleteness of the anterior CoW correlates with a higher frequency of deep lacunes [11]. A greater WMH burden is found in those with an incomplete CoW [9]. Fetal configuration of posterior CoW is associated with a decreasing load of small- and medium-size of deep WMH (DWMH), but not large WMH in patients with clinical manifestations of atherosclerotic disease [12].

Because of the close relationship between CoW morphology, hypertension, and etiology-associated CSVD features, where deep lacunes are more frequent in HA-ICH [13], and the prevalence of multiple subcortical spots of WMH (DWMH) is higher in CAA-ICH [14]; there might be a potential relationship between CoW morphology and ICH etiology. In our present study, we addressed the issue of whether CoW morphology is associated with ICH etiology by comparing the CoW morphology among HA-, CAA-, and mixed-ICH, and whether it is associated with CSVD burden, individually and incorporated in the combined CSVD burden.

## Methods

### Patient Recruitment

We performed a retrospective analysis based on our ongoing longitudinal database collected at the West China Hospital of Sichuan University. The present study subjects were consecutive patients with a diagnosis of primary ICH who had a brain MRI to assess CSVD burden admitted to our center between March 2012 and January 2021. Patients with intracerebral hemorrhage because of hemorrhagic transformation of an ischemic infarct, brain tumor, rupture of a structural vascular lesion such as aneurysm or arteriovenous malformation, or systemic diseases such as liver cirrhosis and renal failure were not enrolled. In the final analysis, we excluded those who had history of ischemic stroke, were diagnosed as primary intraventricular hemorrhage, aged < 18 years at stroke onset, or did not have brain CTA or MRA for CoW assessment. This study was approved by the Ethics Committee on Biomedical Research, West China Hospital of Sichuan University (number of approval document: 362th of Year 2019). The ethics committee waived the need for patient consent for the present study because of the retrospective chart review.

### Definitions of CoW Morphology

**Fig. 1:**
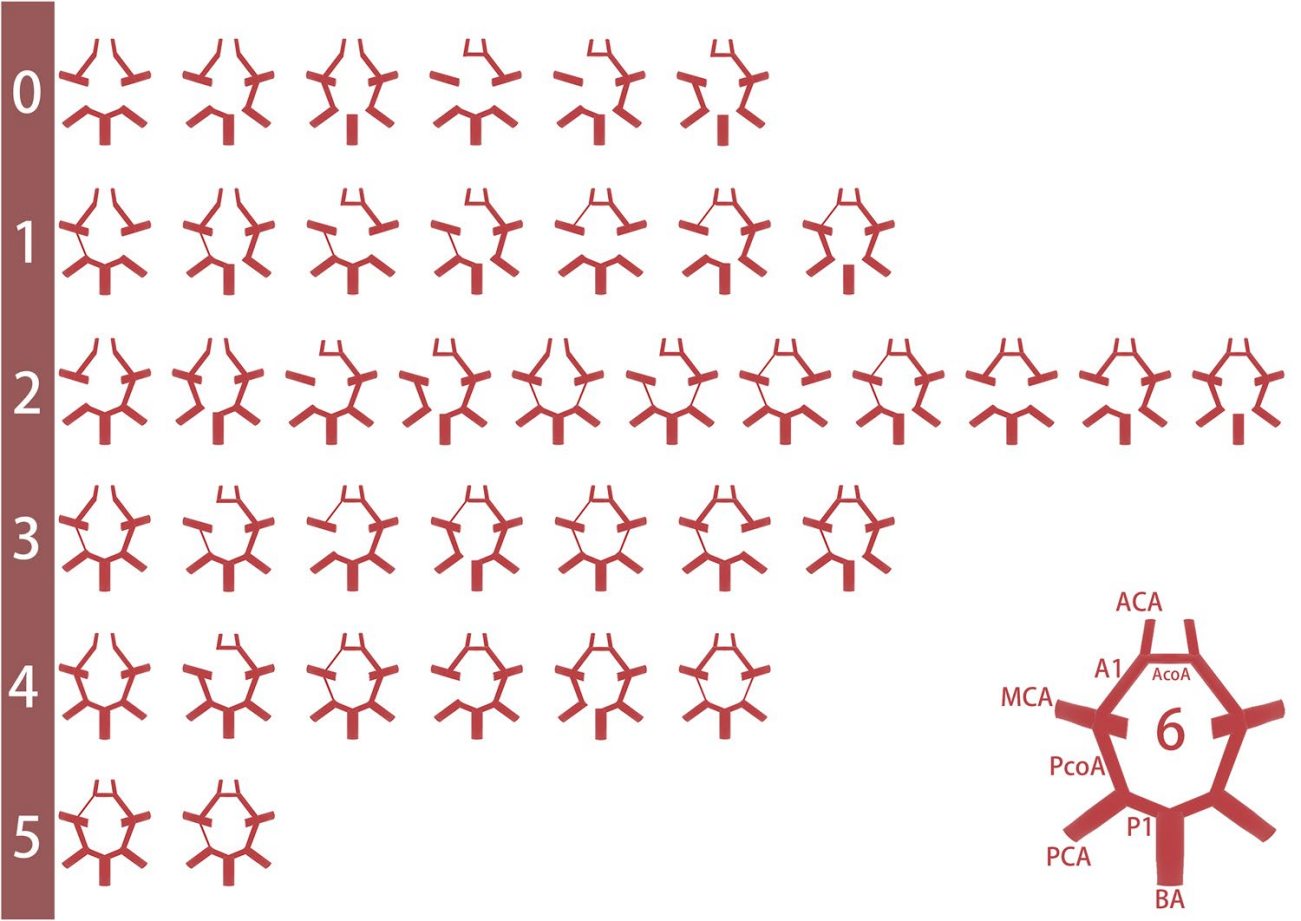
Examples of the CoW score (range 0–6) according to Kim et al.

Two experienced neurologists (Mangmang Xu and Qian Wu) masked to clinical characteristics and ICH etiology reviewed brain CTA or MRA to assess the CoW independently and the disagreements were resolved through discussion. The median time from onset to MRA/CTA was 1.8 (0.5–8.9) days. The presence, diameter, and length of all component vessels of the CoW were evaluated on the source images of 3D time-of-flight and maximum intensity projection images of MRA [9, 15] or source data of CTA [16]. Any vessel that was at least 0.8 mm in diameter was considered as present [15]. In our present study, an incomplete CoW was considered when any vessel of the CoW was absent or less than 0.8 mm in diameter in line with reference standards [15]. We used CoW score according to the score proposed by Kim et al. [17] to evaluate the extent of collateral circulation. CoW score was an ordinal scale ranging from 0 to 6, with a formula of anterior communicating artery (AcoA) score + left posterior communicating artery (PcoA) score + right PcoA score. AcoA score was scored 0 when the AcoA or unilateral A1 segment of the anterior cerebral artery was absent; scored 1 when patients had intact AcoA but A1 diameter was ≤ 50% of that of the contralateral A1; and scored 2 when the AcoA was intact and both A1 diameters were > 50% of that of the contralateral A1. For the PcoA score, 0 points were allocated when the PcoA or P1 segment of the posterior cerebral artery was absent; 1 point when the diameter of PcoA was ≤ 50% of that of the ipsilateral P1; and 2 points when the diameter of PcoA was > 50% of that of the ipsilateral P1 [17]. Figure 1 shows the examples of this CoW score. A poor CoW was defined as a CoW score of 0–2 per a prior study [17]. The complete fetal variant of the PcoA (CFPcoA) was defined when patients had visible PcoA but without P1 [18].

### MRI-Based CSVD Scores

MRIs were performed on a 3.0 T MRI machine. All included subject included subjects had axial T1-, T2-weighted images, susceptibility-weighted imaging (SWI), and fluid-attenuated inversion recovery. The details for MRI parameters were described in our previous study [19]. The median time from symptom onset to SWI was 5.5 (3.1–15.8) days. MRI CSVD features were rated per the STRIVE consensus criteria [2]. We assessed the presence, number, and severity of lacunes, WMH, EPVS, CMBs, and cSS.

According to a previously described and validated ordinal scores [8, 20, 21], we rated global CSVD burden (0–6), total HA burden (0–4), and total CAA burden (0–6) for each included patient. In brief, global CSVD burden was rated by allocating 1 point for the presence of lacunes, 1–4 CMBs, EPVS in BG > 20, and moderate WMH (DWMH + periventricular WMH [PWMH] Fazekas score 3–4), respectively; and 2 points for ≥ 5 CMBs, and severe WMH (DWMH + PWMH Fazekas score 5–6), respectively [8, 21]. We evaluated the total HA burden by allocating 1 point for the presence of (a) lacunes; (b) ≥ 1 deep CMB; (c) EPVS in basal ganglia (BG) > 10; and (d) the presence of WMH (DWMH Fazekas score 2–3 or PWMH Fazekas score 3) [8, 21]. Finally, the total CAA burden was evaluated by accounting 1 point for (a) 2–4 lobar CMBs; (b) EPVS in centrum semiovale (CSO) > 20; (c) the presence of WMH; and (d) focal cSS (restricted to 1–3 sulci); and 2 points for (a ) ≥ 5 lobar CMBs; and (b) disseminated cSS (diffuse to ≥ 4 sulci) [8, 20].

### ICH Etiologic Classification

For all included patients, the ICH etiology was classified as HA-, CAA, mixed-ICH, and undetermined type according to previous studies [13, 14, 22, 23]. HA-ICH was considered when the hemorrhage was located in deep structures (BG, thalamus, and brain stem), with or without deep CMBs, but no lobar CMBs [13, 14]. CAA-ICH was defined when patients had a lobar hemorrhage (cerebellar hemorrhage allowed), aged ≥ 55 years, with or without lobar CMBs or cSS, but no deep CMBs per the modified Boston criteria for probable, or possible CAA [22]. When patients had hemorrhage/CMBs which were located in both deep and lobar regions, they were classified as having mixed-ICH [23]. For those who had strictly lobar ICH, without deep CMBs, but aged < 55 years, they were considered as undetermined type. According to the modified Boston criteria [22], lobar ICH/CMB which was used to define ICH etiology included cerebellar location in the present study.

## Statistical Methods

Clinical and neuroimaging characteristics among HA-ICH, CAA-ICH, and mixed ICH were compared, using ANOVA, Kruskal-Wallis H test, Pearson χ2, and Fisher exact tests, when appropriate. We used univariable and multivariable logistic regression to look for the associations for CoW morphology, including ICH etiology, each CSVD features, and three combined CSVD burden. The association between CoW morphology and ICH etiology was adjusted for age, sex, hypertension history, diabetes mellitus (DM) history, smoking, and ICH location (classified as deep, lobar, or cerebellar). Exploratory analyses in subgroups with age and hypertension history were conducted on the basis that both variates were risk factors for both CoW and ICH [24, 25]. For the association between CoW morphology and CSVD burden, we adjusted for hypertension history, DM history, hyperlipidemia, smoking, and heart disease, as well as age and sex in the multivariable analyses. All tests were performed in IBM SPSS software (version 23). *P*-values < 0.05 were considered to be statistically significant.

## Results

### General Population

Among the 296 enrolled patients who had brain MRIs and a diagnosis of primary ICH, we excluded 2 patients aged < 18 years, 70 without CTA/MRA, 8 with prior ischemic stroke, and 1 with the diagnosis of primary intraventricular hemorrhage. Finally, 215 subjects were included into analysis. Overall, there were 94 HA-ICHs, 28 CAA-ICHs, 89 mixed-ICHs, and 4 undetermined types. The demographic, clinical, and imaging characteristics of the overall population and subgroups by ICH etiology are presented in Table 1. CAA patients were the oldest among HA-, CAA-, and mixed ICH (*p* < 0.001), and mixed ICH had the highest CSVD burden (*p* < 0.001 for each of the three CSVD burden) on univariate analyses.

**Table 1:** Clinical and neuroimaging characteristics by ICH etiology

	Total (*n* = 215)	HA-ICH (*n* = 94)*	CAA-ICH (*n* = 28)*	Mixed ICH (*n* = 89)*
Age, mean (SD), y	60.3 (13.4)	55.2 (12.5)	71.1 (8.6)	63.1 (12.5)**†**
Male, *n* (%)	157 (73.0)	69 (73.4)	19 (67.9)	66 (74.2)
Hypertension history, *n* (%)	141 (65.6)	59 (62.8)	15 (53.6)	66 (74.2)
DM history, *n* (%)	20 (9.3)	7 (7.4)	5 (17.9)	8 (9.0)
Hyperlipidemia, *n* (%)	8 (3.7)	1 (1.1)	0	6 (6.7)
Alcohol, *n* (%)	45 (20.9)	23 (24.5)	2 (7.1)	18 (20.2)
Smoking, *n* (%)	67 (31.2)	28 (29.8)	9 (32.1)	28 (31.5)
CSVD burden				
Global CSVD burden, median, (IQR)	3 (1–4)	1 (0–3)	2 (1–4)	4 (3–5.5)**†**
Total HA burden, median, (IQR)	2 (1–3)	1 (1–2)	1 (1–2)	3 (2–4)**†**
Total CAA burden, median, (IQR)	1 (0–3)	1 (0–1)	2 (1–4)	3 (2–4)**†**
CoW parameters				
Left A1 diameter, mean (SD), mm	2.6 (0.6)	2.6 (0.5)	2.6 (0.5)	2.5 (0.6)
Left A1 length, mean (SD), mm	14.0 (2.2)	14.2 (2.1)	14.1 (2.2)	13.8 (2.3)
Right A1 diameter, mean (SD), mm	2.4 (0.6)	2.4 (0.6)	2.4 (0.6)	2.5 (0.5)
Right A1 length, mean (SD), mm	14.5 (2.1)	14.3 (2.3)	14.3 (2.2)	14.8 (1.8)
AcoA diameter, mean (SD), mm	1.6 (0.5)	1.6 (0.5)	1.4 (0.5)	1.7 (0.6)
Left P1 diameter, mean (SD), mm	2.5 (0.6)	2.4 (0.6)	2.5 (0.5)	2.5 (0.5)
Left P1 length, mean (SD), mm	7.8 (2.2)	7.5 (2.2)	8.1 (1.9)	8.1 (2.3)
Left PcoA diameter, mean (SD), mm	1.7 (0.7)	1.7 (0.6)	1.6 (0.8)	1.7 (0.6)
Left PcoA length, mean (SD), mm	8.2 (2.8)	8.4 (2.5)	6.3 (3.6)	8.3 (2.8)
Right P1 diameter, mean (SD), mm	2.5 (0.6)	2.4 (0.7)	2.4 (0.6)	2.5 (0.5)
Right P1 length, mean (SD), mm	7.2 (2.7)	7.4 (2.6)	8.3 (3.1)	6.6 (2.4)
Right PcoA diameter, mean (SD), mm	1.9 (0.7)	1.9 (0.7)	1.7 (0.7)	2.1 (0.6)
Right PcoA length, mean (SD), mm	9.4 (2.7)	9.2 (2.5)	9.1 (2.5)	9.4 (3.1)
CoW score, median, (IQR)	3 (2–4)	3 (2–4)	2 (2–4)	3 (2–4)
Poor CoW, *n* (%)	102 (47.4)	41 (43.6)	16 (57.1)	43 (48.3)
Incomplete CoW, *n* (%)	161 (74.9)	68 (72.3)	24 (85.7)	67 (75.3)
CoW category				
Complete CoW, *n* (%)	54 (25.1)	26 (27.7)	4 (14.3)	22 (24.7)
Incomplete anterior CoW only, *n* (%)	3 (1.4)	2 (2.1)	0	1 (1.1)
Incomplete posterior CoW only, *n* (%)	122 (56.7)	50 (53.2)	21 (75.0)	50 (56.2)
Incomplete anterior + posterior CoW, *n* (%)	36 (16.7)	16 (17.0)	3 (10.7)	16 (18.0)
CFPcoA, *n* (%)	22 (10.2)	7 (7.4)	2 (7.1)	13 (14.6)

*Four patients with lobar ICH, without CMB, and aged < 55 years were classified as undetermined type and were not included into this analysis. **†** *p* < 0.001 among groups. Abbreviation: *ICH*, intracerebral hemorrhage; *HA*, hypertensive arteriopathy; *CAA*, cerebral amyloid angiopathy; *SD*, standard deviation; *DM*, diabetes mellitus; *CSVD*, cerebral small vessel disease; *IQR*, interquartile range; *CoW*, Circle of Willis; *AcoA*, anterior communicating artery; *PcoA*, posterior communicating artery; *CFPcoA*, complete fetal-variant of PcoA

### The Association Between ICH Etiology and CoW Morphology

**Fig. 2:**
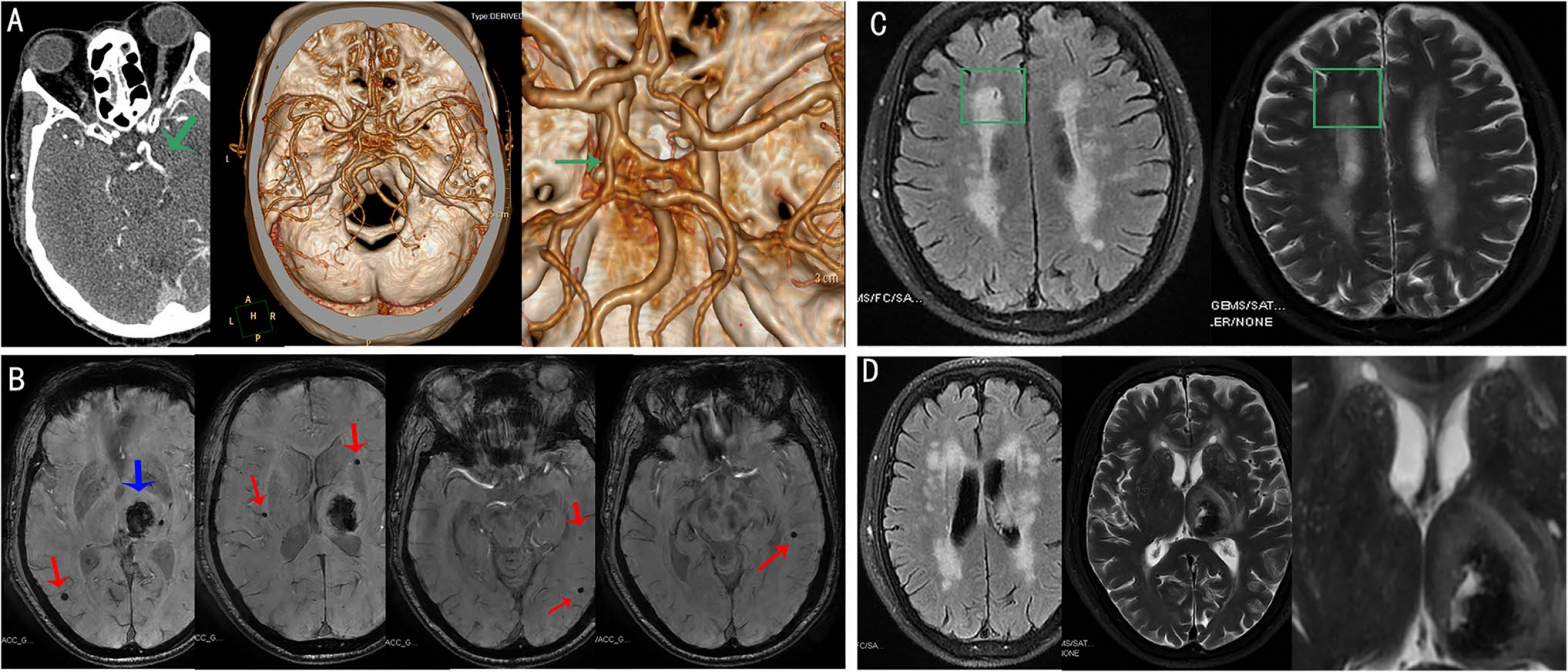
Representative case of mixed ICH without hypertension history had a fetal-variant of PcoA. **A** The fetal-variant of CoW (green arrow); **B** mixed CMB/ICH in SWI sequence (the red arrows show multiple CMBs, and the blue arrow shows an ICH in deep); **C** one lacune in FLAIR and T2-weighted sequences (green box); **D** severe WMH in both deep and periventricular regions and EPVS > 20 in BG

Tables 1 and 2 represent the CoW parameters and morphology in the general population and by ICH etiology. Overall, there were no significant differences in the diameter and length of A1, P1, and PcoA, as well as in CoW score, the presence of poor CoW, incomplete CoW, and CFPcoA. When stratified by age and hypertension, we found that a poor CoW indicated lower incidence of HA-ICH (*p* = 0.050) in patients aged ≥ 60 years. While in patients aged < 60 years, a poor CoW was associated with a higher incidence of HA-ICH (*p* = 0.043), and lower incidence of mixed ICH (*p* = 0.044) (Table 2). After correction for age, sex, hypertension history, DM history, smoking, and ICH location, the differences in subgroup analyses of ≥ 60 years and < 60 years were not significant (data not shown). Overall, there was a trend towards a greater incidence of mixed ICH in patients with CFPcoA compared with those without CFPcoA (59.1% vs 40.2%, *p* = 0.090). In the subgroup of patients without hypertension, the difference reached statistical significance (71.4% vs 28.1%, *p* = 0.032), even after adjusting for age, sex, DM history, smoking, and ICH location (*OR* 17.027, 95% *CI* 1.564–185.356, *p* = 0.020). Figure 2 shows a representative case without hypertension history had a mixed ICH and fetal variant type of PcoA. There were no significant differences between ICH etiology and both poor CoW and incomplete CoW when stratified by hypertension.

**Table 2:** The association of ICH etiology with CoW stratified by age and hypertension*

	Non-noor CoW	Poor CoW	*p*-value	Complete CoW	Incomplete CoW	*p*-value	Non-CFPcoA	CFPcoA	*p*-value
≥60 years (*n* = 114)									
HA, n (%)	18 (35.3)	12 (19.0)	*0.050*	6 (33.3)	24 (25.0)	0.560	28 (28.3)	2 (13.3)	0.347
CAA, *n* (%)	10 (19.6)	15 (23.8)	0.590	2 (11.1)	23 (24.0)	0.353	23 (23.2)	2 (13.3)	0.516
Mixed, *n* (%)	23 (45.1)	36 (57.1)	0.201	10 (55.6)	49 (51.0)	0.725	48 (48.5)	11 (73.3)	0.073
< 60 years (*n* = 97)									
HA, *n* (%)	35 (58.3)	29 (78.4)	0.043	20 (58.8)	44 (69.8)	0.274	59 (65.6)	5 (71.4)	1.000
CAA, *n* (%)	2 (3.3)	1 (2.7)	1.000	2 (5.9)	1 (1.6)	0.280	3 (3.3)	0	1.000
Mixed, *n* (%)	23 (38.3)	7 (18.9)	0.044	12 (35.3)	18 (28.6)	0.494	28 (31.1)	2 (28.6)	1.000
With hypertension history (*n* = 140)									
HA, *n* (%)	29 (45.3)	30 (39.5)	0.486	13 (44.8)	46 (41.4)	0.742	53 (42.4)	6 (40.0)	0.859
CAA, *n* (%)	6 (9.4)	9 (11.8)	0.638	2 (6.9)	13 (11.7)	0.736	14 (11.2)	1 (6.7)	1.000
Mixed, *n* (%)	29 (45.3)	37 (48.7)	0.691	14 (48.3)	52 (46.8)	0.891	58 (46.4)	8 (53.3)	0.611
Without hypertension history (*n* = 71)									
HA, *n* (%)	24 (51.1)	11 (45.8)	0.677	13 (56.5)	22 (45.8)	0.399	34 (53.1)	1 (14.3)	0.107
CAA, *n* (%)	6 (12.8)	7 (29.2)	0.112	2 (8.7)	11 (22.9)	0.199	12 (18.8)	1 (14.3)	1.000
Mixed, *n* (%)	17 (36.2)	6 (25.0)	0.341	8 (34.8)	15 (31.3)	0.766	18 (28.1)	5 (71.4)	0.032†

*Four patients with lobar ICH, without CMB, and aged < 55 years were classified as undetermined type and were not included into this analysis. **†** The association between fetal-variant of PcoA and mixed ICH in patients without hypertension was still significant after correction for age, sex, diabetes mellitus history, smoking, and ICH location (*OR* 17.027, 95% *CI* 1.564–185.356, *p* = 0.020). Abbreviation: *HA*, hypertensive arteriopathy; *CAA*, cerebral amyloid angiopathy; *CoW*, Circle of Willis; *PcoA*, posterior communicating artery; *CFPcoA*, complete fetal variant of PcoA

### CoW Morphology and CSVD Burden

Our data suggested that an incomplete CoW was associated with higher incidence of lacunes (*OR* 2.020, 95% *CI* 1.043–3.913, *p* = 0.037) and CMBs ≥ 5 (*OR* 2.426, 95% *CI* 1.208–4.872, *p* = 0.013), but not with WMH burden, EPVS > 20 in BG, EVPS > 20 in CSO, and cSS. As expected, the combined CSVD burden was higher in patients with an incomplete CoW (*OR* 1.198, 95% *CI* 1.015–1.413, *p* = 0.032 for global CSVD burden; *OR* 1.304, 95% *CI* 1.031–1.651, *p* = 0.027 for total HA burden; *OR* 1.353, 95% *CI* 1.069–1.713, *p* = 0.012 for the total CAA burden). Adjustment for hypertension history, DM history, hyperlipidemia, smoking, and heart disease did not alter the direction or strength of the association of incomplete CoW with lacune (adjusted *OR* 2.114, 95% *CI* 1.062–4.207, *p* = 0.033), CMB ≥ 5 (adjusted *OR* 2.437, 95% *CI* 1.187–5.002, *p* = 0.015), global CSVD burden (adjusted *OR* 1.194, 95% *CI* 1.004–1.419, *p* = 0.045), and the total CAA burden (adjusted *OR* 1.343, 95% *CI* 1.056–1.707, *p* = 0.016). However, after additional adjustment for age and sex, these associations were no longer significant (Table 3).

**Table 3:** The association between CSVD markers and incomplete CoW (*n* = 215)

	Univariable analysis		Multivariable analysis (model 1)		Multivariable analysis (model 2)	
	*OR* (95% *CI*)	*p*-value	Adjusted OR (95% *CI*)	*p*-value	Adjusted *OR* (95% *CI*)	*p*-value
Any Lacune	2.020 (1.043–3.913)	***0.037***	2.114 (1.062–4.207)	0.033	1.802 (0.883–3.677)	0.106
CMB ≥ 1	1.149 (0.589–2.241)	0.684	1.038 (0.511–2.110)	0.917	0.903 (0.433–1.884)	0.786
CMBs ≥ 5	2.426 (1.208–4.872)	***0.013***	2.437 (1.187–5.002)	0.015	2.034 (0.967–4.276)	0.061
Presence of lobar CMB	1.375 (0.730–2.592)	0.324	1.337 (0.692–2.582)	0.387	1.061 (0.531–2.117)	0.867
Lobar CMB ≥ 5	2.003 (0.835–4.805)	0.120	2.041 (0.840–4.959)	0.115	1.790 (0.717–4.473)	0.212
Total WMH score	1.136 (0.955–1.352)	0.151	1.102 (0.919–1.321)	0.295	0.974 (0.798–1.189)	0.797
DWMH Fazekas score	1.298 (0.939–1.793)	0.114	1.245 (0.889–1.744)	0.202	1.034 (0.720–1.486)	0.856
PWMH Fazekas score	1.203 (0.867–1.668)	0.269	1.126 (0.799–1.585)	0.498	0.877 (0.600–1.283)	0.499
The presence of WMH	1.631 (0.870–3.057)	0.127	1.537 (0.798–2.962)	0.199	1.078 (0.532–2.182)	0.836
EPVS in BG > 20	1.421 (0.700–2.883)	0.331	1.385 (0.668–2.872)	0.381	0.709 (0.301–1.669)	0.431
EPVS in CSO > 20	1.761 (0.917–3.383)	0.089	1.668 (0.856–3.253)	0.133	1.340 (0.668–2.688)	0.410
cSS presence	1.731 (0.717–4.179)	0.222	1.865 (0.754–4.615)	0.177	1.187 (0.452–3.118)	0.727
Disseminated cSS	1.369 (0.371–5.046)	0.637	1.531 (0.401–5.847)	0.533	0.919 (0.226–3.743)	0.906
Global CSVD burden	1.198 (1.015–1.413)	0.032	1.194 (1.004–1.419)	0.045	1.078 (0.892–1.302)	0.439
Total HA burden	1.304 (1.031–1.651)	0.027	1.282 (0.997–1.648)	0.053	1.134 (0.867–1.483)	0.359
Total CAA burden	1.353 (1.069-1.713)	0.012	1.343 (1.056-1.707)	0.016	1.193 (0.925-1.538)	0.173

Model 1: adjusted for hypertension history, diabetes mellitus history, hyperlipidemia, smoking, and heart disease. Model 2: adjusted for factors in model 1 + age and sex. Abbreviation: *CSVD*, cerebral small vessel disease; *CoW*, Circle of Willis; *OR*, odds ratio; *CI*, confidence interval; *CMB*, cerebral microbleed; *WMH*, white matter hyperintensities; *DWMH*, deep WMH; *PWMH*, periventricular WMH; *BG*, basal ganglia; *CSO*, centrum semiovale; *cSS*, cortical superficial siderosis; *HA*, hypertensive arteriopathy; *CAA*, cerebral amyloid angiopathy

Poor CoW was not significantly associated with each CSVD feature or each combined CSVD burden either in the general population, or in subgroups stratified with age with a cut off 60 years, or hypertension history (data not shown). Although the presence of CFPcoA did not affect the individual CSVD feature or combined CSVD burden overall (data not shown), fetal variant of PcoA in subgroup of patients without hypertension was significantly associated with high incidence of lacune (*OR* 13.143, 95% *CI* 1.487–116.152, *p* = 0.021), CMB ≥ 5 (*OR* 6.806, 95% *CI* 1.211–38.254, *p* = 0.029), global CSVD burden (*OR* 1.814, 95% *CI* 1.141–2.884, *p* = 0.012), total HA burden (*OR* 2.282, 95% *CI* 1.152–4.520, *p* = 0.018), and total CAA burden (*OR* 1.897, 95% *CI* 1.183–3.043, *p* = 0.008). Figure 2 also shows the representative case without hypertension history had a fetal variant type of PcoA and high CSVD burden. After correction for DM history, hyperlipidemia, smoking, and heart disease, as well as age and sex, the fetal variant of PcoA still affected the global CSVD burden (adjusted *OR* 2.025, 95% *CI* 1.026–3.997, *p* = 0.042), total HA burden (adjusted *OR* 3.569, 95% *CI* 1.106–11.513, *p* = 0.033), and total CAA burden (adjusted *OR* 2.626, 95% *CI* 1.140–6.053, *p* = 0.023), but not lacune or CMB ≥ 5. However, in patients with hypertension, these associations were not significant (data not shown).

## Discussion

The CoW is often investigated in patients with atherosclerotic disease or ischemic stroke [12, 16, 17, 25], but little attention has been given to patients with ICH in the literature. Our present study in consecutive ICH patients finds that in patients aged < 60 years, poor CoW was associated with a higher incidence of HA-ICH, and a lower incidence of mixed ICH, although the significant associations were not independent. It is well-known that patients with HA-ICH were often younger than CAA-ICH patients [14]. And data from our present study and previous reports [23, 26] indicated that patients with mixed-ICH were older than those with HA-ICH, but younger than patients with CAA-ICH. Therefore, the higher incidence of HA-ICH in patients with poor CoW in those with younger age is expected. The impact of poor CoW in ICH etiology might be indirect. Vrselja et al. [27] have pointed out that CoW and its communicating arteries protect cranial microvasculature and blood–brain barrier by functioning as a pressure absorber mechanism (i.e., receive great blood pressure burden) in high blood pressure condition. In addition, penetrating arterioles in deep brain regions, which have stiff and brittle structure due to the degeneration of focal smooth muscle cell and collagen deposition in the vessel walls, are prone to burst because of their proximity to the CoW [28]. Therefore, the poor CoW, which might partly lose the ability to absorb great pressure, could lead to deteriorate the stiff and brittle penetrating arterioles which lie downstream of the CoW, and then bring into an ICH prone situation. However, this assumption needs to be verified in future studies.

The presence of CFPcoA was significantly associated with greater incidence of mixed ICH in patients without hypertension, independent of age, sex, DM history, smoking, and ICH location. Interestingly, fetal variant of PcoA also affected CSVD burden, with higher presence of CFPcoA, more severe global CSVD burden, total HA burden, and total CAA burden, even after adjusting for age, sex, and vascular risk factors. As shown in Table 1 in our present study and previous studies [23, 26], mixed ICH had the highest CSVD burden except for cSS which is a MRI marker for CAA by the modified Boston criteria [22]. Therefore, it is reasonable that patients with CFPcoA would more likely to have mixed ICH when compared with HA- or CAA-ICH. Previous studies have shown that in cases with fetal variant of PcoA, vascular insufficiency is more prone to be developed than non-fetal variant of PcoA because of the nature of this anatomy variants [29]. In addition, an ischemia status caused by carotid stenosis ≥ 25% is associated with deep and mixed CMB [30], where patients with mixed CMB were classified as having mixed ICH. Taken together, fetal-variant PcoA indicates a high likelihood of small vessel disease in both deep and lobar regions, which could be caused by severe HA, or the mixture of HA and CAA [23, 26, 31]. Our results indicate that in the absence of a history of hypertension, fetal-variant PcoA might be another important factor in triggering severe CSVD.

Another important finding is the association of incomplete of CoW with CSVD burden in ICH patients. A previous study showed that individuals with incompleteness of the anterior CoW defined as absent A1 segment correlated with a higher frequency of lacunes in healthy individuals [11]. In the present study, we found a higher frequency of lacunes either in patients with incomplete CoW or in patients with incomplete posterior CoW (*OR* 2.257, 95% *CI* 1.170–4.354, *p* = 0.015), but not in those with incomplete anterior CoW. The different result about incomplete anterior CoW and lacune could be explained by the different study population and that the frequency of absent A1 was only 1.9% (4/215). Also, we found that the presence of CFPcoA correlates with a higher presence of lacune, especially in subgroup of patients without hypertension, after correction for age, sex, and vascular risk factors. Our present study expands our understanding of CoW morphology and lacunes in addition to previously published study by Miyazawa et al. [11]. As for WMH, Ryan et al. [9] found a greater WMH burden in subjects with incomplete CoW; however, we did not found any difference between patients with and without incomplete CoW either in total WMH score or in DWMH, while the latter is in accordance with van der Grond et al.’s finding [12]. The main difference between our study and the study by Ryan et al. [9] is that Ryan et al. evaluated WMH in older patients (aged greater than 50 years), and these subjects might have a higher white matter lesion load than ours. Furthermore, the different score systems of WMH could also be an underlying factor that influences the result. In this respect, future studies are needed to investigate the association of CoW morphology with quantitative WMH burden.

Of note, our findings also suggest that most patients with an incomplete CoW or CFPcoA, especially in patients without hypertension history had severe CSVD burden. With regard to the high stroke recurrence and unfavorable functional outcome in ICH patients with severe CSVD burden [6, 8], the variant of CoW has significant clinical meaning that it might have an impact on the mechanisms of ICH, while potentially aiding prevention of stroke onset and stroke recurrence.

Our study had several limitations. We included participants who had brain MRIs, and therefore there might be selection bias. However, MRI, particularly SWI or gradient echo T2* sequence, is useful for detecting CMB or cSS, making it a good choice for careful identification of the ICH mechanisms to optimizing management [4]. Furthermore, MRI is more sensitive to small changes of small vessel diseases [3]. In our present study, we assessed not only individual CSVD features, but also the combined CSVD burden using different weightings to overall reflect the severity of CSVD burden. Second, the sample size was relatively small, resulting in the wide 95% CI in subgroup analyses in patients with and without hypertension. Studies with larger sample size are necessary to confirm our results and to explore the underlying mechanism.

## Conclusions

In summary, our study shows that CoW morphology has an important role in ICH, for instance, CFPcoA affects the occurrence of mixed ICH, and both incomplete CoW and fetal variant of PcoA are associated with high CSVD burden. Our findings are novel, and need to be verified in future independent study. If replicated, our results help to improve our understanding of the mechanisms of primary ICH and have important implications for the risk assessment for mixed ICH.

## Data Availability

Data will be made available on reasonable request.
